# Health literacy: psychometric properties of the HLS-EU-Q16 questionnaire in a Peruvian adult population

**DOI:** 10.3389/fpubh.2026.1762195

**Published:** 2026-02-05

**Authors:** Daniel F. Condor-Camara, Judith Francisco-Pérez, Gabriel Ortiz-Francisco, Zoila Esperanza Leiton-Espinoza, Johnny R. Jurado Boza, Nancy Ana Coaquira Machaca, Katherine L. Sánchez-Álvarez, Yuly S. Hilario-Pizarro, Jacqueline Begazo Corahua, Nataly Ventura-Román

**Affiliations:** 1 Faculty of Nursing, Universidad Peruana Cayetano Heredia, Lima, Peru; 2 Faculty of Health and Wellbeing, Pontificia Universidad Católica del Ecuador, Quito, Ecuador; 3 Faculty of Psychology, Universidad Técnica Particular de Loja, Loja, Ecuador; 4 Faculty of Nursing, Universidad Nacional de Trujillo, Trujillo, Peru; 5 Faculty of Nursing, Universidad Peruana Los Andes, Huancayo, Peru; 6 Faculty of Nursing, Universidad Nacional de San Agustin de Arequipa, Arequipa, Peru; 7 Faculty of Medicine, Postgraduate School, Universidad Nacional Mayor de San Marcos, Lima, Peru; 8 Faculty of Health Sciences, Universidad Nacional Daniel Alcides Carrion, Tarma, Peru; 9 Challhuani Health Center, Regional Health Directorate of Apurimac, Challhuani, Apurimac, Peru

**Keywords:** global health, health education, health literacy, primary health care, public health

## Abstract

**Background:**

Health literacy is a key component of public health because it is related to quality of life, chronic disease management, and trust in health services, yet it must be measured with reliable instruments.

**Aim:**

To evaluate the validity and reliability of the Health Literacy Survey European Questionnaire’s short version (HLS-EU-Q16) in Peruvian adults and to analyze health literacy according to sociodemographic variables.

**Methods:**

A cross-sectional psychometric validation study was conducted with 438 participants aged 18 to 80 years using convenience sampling. The culturally adapted HLS-EU-Q16 was administered online; its psychometric properties were analyzed using confirmatory factor analysis and reliability coefficients. Differences in literacy according to sociodemographic characteristics were evaluated using ANOVA and Pearson’s correlations.

**Results:**

Confirmatory factor analysis confirmed the three-dimensional structure (health care, disease prevention, and health promotion) with excellent fit indices (CFI = 0.99, TLI = 0.99, RMSEA = 0.02, SRMR = 0.04). Internal consistency was high (α = 0.95; ω = 0.96). Significant differences in health literacy were found according to region (coast > highlands, *p* < 0.001), educational level (higher education > primary education, *p* < 0.001), income (higher income > lower income, *p* < 0.001), and access to health services (shorter travel time > longer travel time, *p* < 0.001). Health literacy decreased with age (*r* = −0.13, *p* < 0.001) and was higher in people with greater access and economic stability. No significant differences were observed by sex or ethnicity.

**Conclusion:**

The HLS-EU-Q16 demonstrates excellent validity and reliability for assessing health literacy in Peru. Significant health-literacy inequalities were identified, influenced by education, income, and access to services. These findings highlight the need for targeted strategies to improve health literacy and reduce disparities in access to and use of health information.

## 1 Introduction

Health literacy (HL) is a relevant variable in public health and occupies a central position in health research. It encompasses the personal characteristics and social resources that individuals need to access, understand, evaluate, and apply health information and services to make decisions about their health or health-related issues (1). Several studies have shown that HL is associated with quality of life (2), positively influences chronic disease management (3), and acts as a mediator in building trust in health services (4).

Health literacy is influenced by several factors that affect individuals’ ability to access and understand health information. These include limited access to reliable information, lack of educational resources, absence of effective health-promotion programs, and the low quality of information available in digital and traditional media (5).

Cultural and linguistic barriers also significantly impact HL. Ethnic minorities, non-native speakers, and those with lower educational levels often encounter difficulties accessing relevant health information (6, 7). These difficulties are further exacerbated by cultural beliefs that may conflict with conventional medical practices, resulting in mistrust and, in some cases, causing individuals to disregard the recommendations of health-care professionals (8, 9). Research has demonstrated that individuals with low HL experience higher hospitalization rates and poorer health outcomes (10).

Education, socioeconomic status, and access to healthcare services influence HL and individuals’ ability to process and apply information (11, 12). Poverty and lack of education perpetuate inequalities and hinder participation in preventive activities, understanding medical indications, and adherence to treatment. These issues have a detrimental impact on both individual and public health.

Studies conducted in Peru have investigated HL among various groups, including hypertensive patients (13), hospitalized individuals (14), patients with chronic conditions receiving primary care (15), individuals vaccinated against COVID-19 (16) and tuberculosis patients (17). These studies found inadequate literacy levels ranging from 24 to 40%. Most of these studies (13, 14, 16, 17) employed the Short Assessment of Health Literacy for Spanish-speaking Adults (SAHLSA-50), which focuses primarily on reading comprehension and medical terminology recognition rather than the broader multidimensional conceptualization of HL encompassing access, understanding, appraisal, and application across healthcare, disease prevention, and health promotion domains. One study (15) used the HLS-EU-Q16 but did not report psychometric validation in the Peruvian context. In contrast, HL was above 54% in the general population (18), although this study used a non-validated Spanish adaptation of the HLS-EU-Q47 administered to university students at a single institution (Universidad Nacional del Centro del Perú), which limits its generalizability to the broader, more diverse Peruvian adult population. The higher literacy levels in this sample reflect the educational background of university students rather than patterns of the general population. This gap in validated, comprehensive HL measurement tools for Peru underscores the need for the present validation study.

Assessing HL is crucial for understanding population characteristics and designing effective intervention strategies. While HL can be measured using multiple methods, one of the most recognized is the European Health Literacy Survey Questionnaire (HLS-EU-Q) (12), which consists of two sections: a core health literacy section and a section addressing the determinants and outcomes associated with HL. Both HLS-EU-Q47 and its short version HLS-EU-Q16 (19) aim to measure individuals’ abilities to access, understand, appraise, and apply health information in three domains: healthcare, disease prevention, and health promotion.

The HLS-EU-Q47 model (20) uses a framework with twelve domains, including four information-processing areas (accessing, understanding, appraising, and applying) and three health domains (healthcare, disease prevention, and health promotion), allowing comparisons within and between countries. The HLS-EU-Q16 has shown inconsistencies in different contexts – for example, Spain (21) and Sweden (7) identified a single factor; in Mexico, four items were removed, yielding two factors (22). In other countries such as Portugal (23), the original structure was maintained, but studies in Sweden (24) and Iceland (9) identified additional factors that do not align with the proposed model. These variations show the lack of a universally defined and validated factor structure, highlights the need to conduct measurement invariance studies across language versions to avoid bias in estimates of internal consistency (Cronbach’s alpha).

This study aims to examine the psychometric properties of the HLS-EU-Q16 in the adult Peruvian population (6, 9, 21, 25). Although this brief and user-friendly instrument has shown variable psychometric performance across countries and cultural contexts, evidence regarding its validation in Peru remains limited. Therefore, assessing its reliability and validity in Peruvian adults is warranted to support its appropriate use in this setting. Accordingly, the present study evaluates the validity and reliability of the HLS-EU-Q16 in a sample of Peruvian adults.

## 2 Materials and methods

### 2.1 Design

A cross-sectional psychometric validation study was conducted using convenience sampling. Data were collected between November 2023 and February 2024 through a self-administered online survey distributed to Peruvian adults aged 18 to 80 years, of all genders. The study aimed to culturally adapt and validate the HLS-EU-Q16 instrument in the Peruvian context by examining its factorial structure, internal consistency, and discriminant validity across sociodemographic groups.

### 2.2 Instrument

The HLS-EU-Q16 (19) was used to measure the health-literacy levels of the population. This instrument consists of sixteen questions and was validated in a Spanish-language version by Nolasco et al. (21). It measures the degree to which people can access, understand, evaluate, and use health-related information to make informed decisions. Each question was answered using a five-point Likert-type scale (Very Easy, Easy, Neutral, Difficult, and Very Difficult) to assess HL across three main domains: healthcare, disease prevention, and health promotion. In addition, sociodemographic questions were included to characterize the participants in terms of age, gender, education level, immigration status, and presence of chronic diseases, thereby providing contextual information about health-literacy levels.

### 2.3 Cultural adaptation

The instrument translated into Spanish was developed by Nolasco (21). To adapt the questions to the local context, the Peruvian research team held a meeting to review each item to ensure its relevance, appropriateness (9, 24), and comprehensibility to the target population. During this process, uncommon terms were identified for replacement or elimination, and more frequently used expressions were incorporated, taking care not to alter the meaning of the original questions. The amendments were approved by consensus or, when this was not possible, by majority vote. Subsequently, a reliability test was conducted on fifty individuals with characteristics similar to those of the target population.

### 2.4 Population and sample

Peru has an estimated population of approximately thirty-four million people, with twenty-five million adults (26). A convenience sampling method was used to recruit 438 participants. Recruitment occurred through systematic approaches in healthcare facilities, shopping centers (department stores), markets, main city squares and other public spaces with high foot traffic across multiple regions of Peru. Research team members approached potential participants in different geographic regions, briefly explained the study objectives, and invited them to participate. Those who expressed interest were provided with a unique link to the online survey, which was shared via messaging apps such as WhatsApp or e-mail, allowing them to complete it at their convenience on their personal devices. Follow-up reminders were not sent to maintain participant autonomy and avoid coercion.

The first section of the survey included informed consent, and upon acceptance, participants proceeded to the HLS-EU-Q16 questionnaire. The online questionnaire took 8–12 min to complete (mean = 10 min, SD ± 2.3). All participants completed the survey independently without structured interviews or assistance. The self-administered online format required participants to possess adequate reading comprehension and digital literacy skills. The survey was configured with mandatory responses for all items to ensure data completeness. Participants could abandon the survey at any point if they chose not to continue; in such cases, no partial data were recorded in the database. Only fully completed questionnaires were automatically saved and included in the analysis, thereby eliminating incomplete data, and ensuring that all 438 participants in the final sample had complete responses.

The convenience sampling approach was selected due to practical constraints related to geographic diversity and accessibility across multiple Peruvian regions. Inclusion criteria required participants to be adults between 18 and 80 years who voluntarily agreed to participate. In the case of older adults, a cognitive screening test was administered to ensure they had adequate cognitive functioning (27).

It is important to acknowledge that this convenience sample is not representative of all Peruvian adults. The recruitment strategy and online survey format overrepresent more educated, urban, and digitally connected participants with better access to technology and internet connectivity. The requirement for independent completion of an online survey inherently excluded individuals with limited reading comprehension, severe cognitive impairment, or insufficient digital literacy. Rural populations, individuals with lower educational levels, and those with limited digital literacy may be underrepresented in this sample. These factors should be considered when interpreting the results and their generalizability to the broader Peruvian population.

### 2.5 Data analysis

Descriptive statistics, including measures of central tendency and dispersion, along with inferential analyses, were performed using SPSS® version 25 for Mac OS. Confirmatory factor analysis (CFA) was conducted using the lavaan package in the R programming environment (version 4.4.1) through RStudio.

The results from the HLS-EU-Q16 scale were analyzed using the mean scores for each item, classifying HL levels into the domains of health care, disease prevention, and health promotion. The following classification was adopted: Insufficient HL: 1–2.5; Moderate HL: 2.6–3.75; Sufficient HL: 3.76–5.

This analytical approach was selected for several reasons supported by psychometric literature. First, mean scores preserve the full range of response variability and provide greater statistical power for detecting differences between groups (28). Second, continuous scores allow for more nuanced interpretations of HL levels and are more appropriate for correlation and regression analyses (29). This approach facilitates the identification of specific areas requiring intervention by maintaining the granularity of responses across the three domains (healthcare, disease prevention, and health promotion).

While this deviates from the original dichotomous scoring recommended by Sørensen et al. (20), it aligns with contemporary psychometric practices for Likert-scale instruments and offers a more detailed, multidimensional perspective on HL in the Peruvian context. To ensure comparability with studies using the original scoring method, we conducted a supplementary analysis using the dichotomous classification which confirmed that the main findings regarding sociodemographic differences remain consistent across both scoring approaches.

A Confirmatory Factor Analysis (CFA) was conducted to assess the three-dimensional model: healthcare, disease prevention, and health promotion (12), proposed by Sorensen et al. (30) and confirmed by Francisco-Pérez et al. (31) in the Venezuelan adult population. The first factor included seven items (items 1, 2, 3, 4, 5, 6, and 7), the second factor included five items (items 8, 9, 10, 11, and 12), and the third factor included four items (item 13, 14, 15, and 16).

Because the questionnaire uses Likert-type items and the data are ordinal, the weighted least squares mean, and variance-adjusted (WLSMV) estimator was employed (29). The following indices were used to assess goodness of fit: the normed χ^2^ (χ^2^/df), comparative fit index (CFI), Tucker-Lewis index (TLI), standardized root mean square residual (SRMR), and root mean squared error of approximation (RMSEA). The criteria used to determine model adequacy were: χ^2^/df ≤ 3 as adequate fit and ≤ 2 as optimal (32); CFI and TLI ≥ 0.90 as adequate and ≥ 0.95 as optimal; RMSEA and SRMR ≤ 0.08 as adequate and ≤ 0.05 as optimal (33).

The reliability of the HL scale and its three domains was evaluated using Cronbach’s alpha and omega coefficients, to measure the internal consistency of the items within each domain and across the total scale (α > 0.80). ANOVA and Student’s t-tests were used to compare HL levels and their three domains across different sociodemographic categories. When significant results were detected, Tukey’s HSD *post hoc* test was used to identify significant differences between groups of variables.

Finally, Pearson’s correlation was used to study the relationships between quantitative variables (age, duration of chronic illness, time as a caregiver) and health literacy levels, as well as with each of the three domains.

### 2.6 Ethical considerations

The study follows the ethical guidelines established by the Declaration of Helsinki and the Belmont Report. Informed consent was obtained from all participants who voluntarily accepted participation in the study. The study was approved by the Ethics Committee of the Universidad Peruana Cayetano Heredia (certificate CIEI-389-36-23).

## 3 Results

### 3.1 Cultural adaptation

The research team reviewed each item of the HLS-EU-Q16 in Spanish and modified five items to improve cultural relevance in Peru. The term “pharmacist” was removed in items 2, 4 and 7 because it is not commonly used in the country. In item 2, the term “psychologist” was added to reflect this professional’s role in healthcare teams. In item 3, the term “health professionals” was used to encompass all healthcare providers in Peru who offer advice, education, and recommendations. Lastly, the term “Pilates” was removed from item 13 as it is not widely recognized in Peru.

The adapted instrument was tested on a sample of 50 participants (28 men and 22 women), aged 18 to 77 years (mean = 35.24; SD ± 16.25). No issues were identified regarding wording or comprehension of the questions. A reliability analysis using Cronbach’s alpha yielded a value of 0.912, indicating extremely high reliability.

### 3.2 Characterization of the sample

The sample was male (55.02%), and most participants residing in the highlands (63.70%) with most representation from the province of Junín (41.78%). The majority of participants considered themselves as mestizos (72.83%) and had complete higher education (48.86%). A substantial proportion owned their homes (67.35%) and did not receive government assistance (93.38%). Over 70% of participants were employed, either as salaried workers (39.50%) or self-employed (36.53%), and the majority worked between 24 and 48 h per week (32.19%) (Table 1).

**Table 1:** Characteristics of the participants.

Variable	Characteristics	Frequency (*n* = 438)	%
Sex	Male	241	55.02
	Female	197	44.98
Region	Highlands	279	63.70
	Coast	131	29.91
	Jungle	28	6.39
Department	Junín	183	41.78
	La Libertad	53	12.10
	Arequipa	51	11.64
	Lima	36	8.22
	Other	115	26.26
Ethnicity	Mestizo	319	72.83
	Quechua	77	17.58
	Caucasian	19	4.34
	Other	23	5.25
Educational level	Superior university	214	48.86
	Technical superior	102	23.29
	High School	101	23.06
	Incomplete Elementary School	21	4.79
Housing	Own	295	67.35
	Rented	143	32.65
Government social support	No	409	93.38
	Yes	29	6.62
Employment status	Dependent worker	173	39.50
	Self-employed	160	36.53
	Unemployed	105	23.97
Working hours per week	Less than 24 h	63	14.38
	24 to 48 h	141	32.19
	More than 48 h	129	29.45
	Unemployed	105	23.97
Time to get to work	Less than 15 min	123	28.08
	15 to 30 min	95	21.69
	More than 30 min	115	26.26
	Unemployed	105	23.97
Financial income	They cover your needs, but you cannot save	237	54.11
	Do not meet their needs	107	24.43
	They meet your needs and can also save you money	94	21.46
Health insurance	SIS	196	44.75
	EsSalud	142	32.42
	None	63	14.38
	Private	37	8.45
Time to go to a health care facility	Less than 15 min	118	26.94
	15 to 30 min	152	34.70
	30 to 45 min	92	21.00
	More than 45 min	76	17.35
Chronic disease	No	386	88.13
	Yes, one	42	9.59
	Yes, more than one	10	2.28
Caregiver of chronically ill family member	No	394	89.95
	Yes, one	34	7.76
	Yes, more than one	10	2.28
Age*		37.76	(18–74)
Time chronic disease*		8.83	(1–53)
Time as caregiver*		7.68	(1–30)

^*^Mean (range).

A considerable proportion of participants commuted to work in less than 15 min (28.08%) and required 15–30 min to reach a health-care facility (34.70%). More than half of the sample reported that their income was sufficient to meet basic needs, yet it did not allow for savings (54.11%). Regarding health-insurance coverage, most participants were enrolled in the government’s Comprehensive Health Insurance program (SIS; 44.75%) followed by EsSalud (32.42%), which covers salaried workers (Table 1).

A large majority of participants reported having no chronic illness (88.13%) and not caring for a family member with one (89.95%; Table 1).

The mean age of participants was approximately 33 years (range: 18–74 years). Participants with chronic illness (11.87%) had lived with their condition for an average of 8.8 years, with a considerable variability (SD = 9.13), and some individuals having been affected for over 50 years. These findings suggest substantial variation in participants’ experiences of chronic illness. Caregivers had served in their roles for an average of 7.7 years (SD = 6.49), indicating considerable variability, with some caring for as many as 30 years (Table 1).

### 3.3 Confirmatory factor analysis

**Figure 1:**
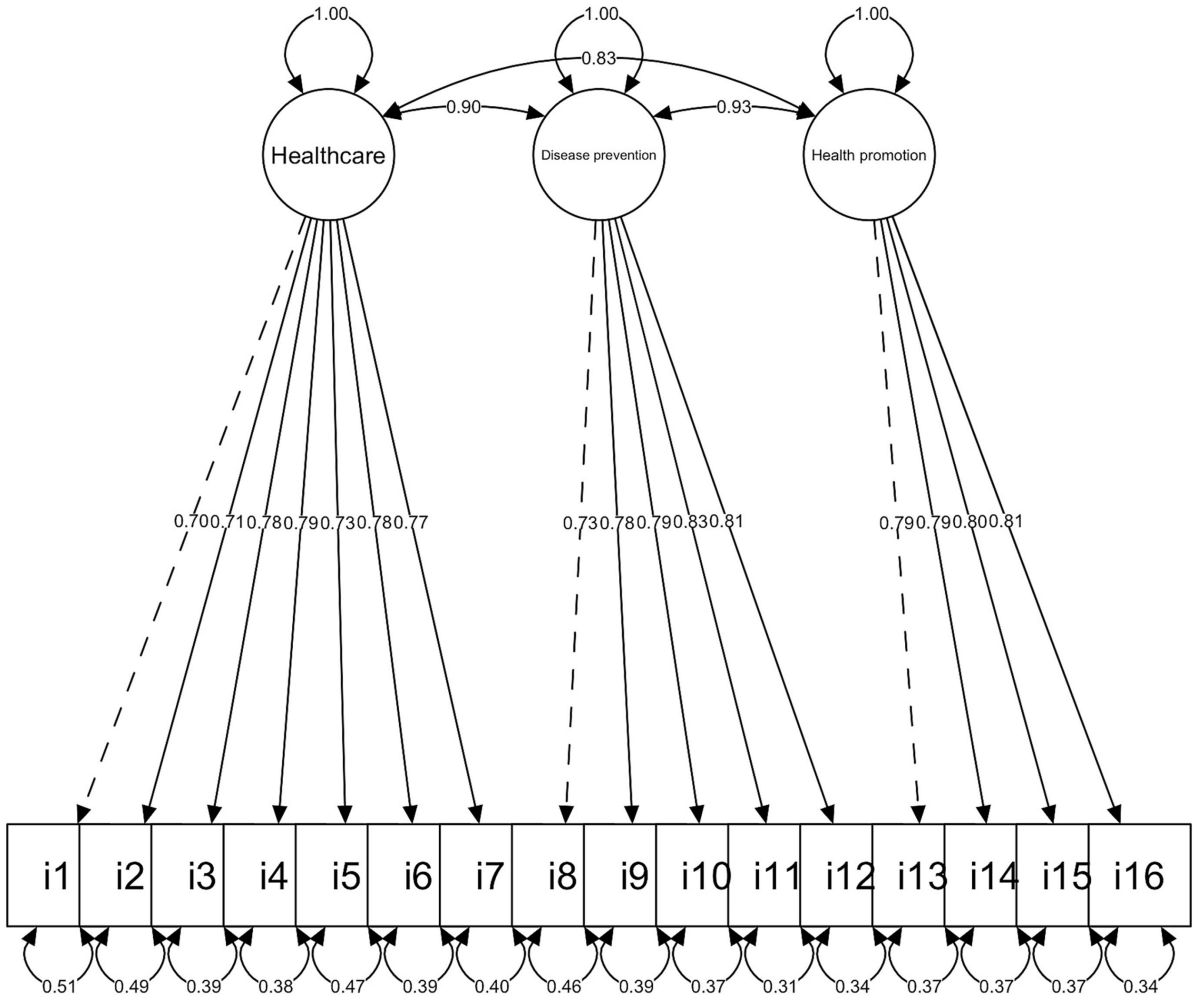
Diagram of the confirmatory factor analysis of the HLS-EU-Q16 Spanish version.

The results suggest a good fit of the three-dimensional model (Table 2). All factor loadings were at or above 0.50, and all three domains were highly correlated (>0.80) (Figure 1).

**Table 2:** Confirmatory factor analysis of the HL scale and its dimensions.

*X^2^*/*df*	≤ 3 appropriate; ≤ 2 optimal	1.67
CFI	≥ 0.90 appropriate; ≥ 0.95 optimal	0.99
TLI		0.99
RMSEA	≤ 0.08 appropriate; ≤ 0.05 optimal	0.03 (0.02–0.03)
SRMR		0.04

### 3.4 Reliability

Reliability analysis using both Cronbach’s alpha and omega coefficients indicated excellent internal consistency (α = 0.95; ω = 0.96) for the overall scale as well as for each of its three domains: healthcare (α = 0.89; ω = 0.93), disease prevention (α = 0.89; ω = 0.91), and health promotion (α = 0.87; ω = 0.89). These results suggest excellent internal consistency (>0.80), which is considered satisfactory (7).

### 3.5 Analysis of health literacy in the sample

Health literacy was measured across the three reported domains. The overall score had a mean of 3.60 (SD = 0.73), reflecting a moderate level of HL with some variability. The health-promotion domain recorded the highest mean score (3.68, SD = 0.78), indicating that participants possessed greater knowledge in this area. Health promotion refers to the ability to access, understand, interpret, and make informed decisions about health determinants within one’s social and physical environment (Table 3).

**Table 3:** Health literacy score by dimension.

Dimensions	Mean	SD	Median	Minimum	Maximum
Health care	3.57	0.74	3.50	1	5
Disease prevention	3.58	0.83	3.60	1	5
Health promotion	3.68	0.78	3.75	1	5
Health Literacy – Total	3.60	0.73	3.67	1	5

In contrast, participants displayed only a moderate level of knowledge and skills regarding disease prevention and healthcare.

### 3.6 Analysis of population characteristics by health literacy and its domains

Statistically significant differences in HL were found based on region (*F* = 7.33; *p* < 0.01) and across all three domains of the scale (Table 4). Post-hoc analysis indicated that these differences were primarily between the coastal and highland regions, with participants from the coast showing significantly higher HL scores (mean = 3.79) compared to those from the highlands (mean = 3.51; *p* < 0.01).

**Table 4:** Characteristics of the population by HL and its dimensions.

Characteristics	Dimensions											
	Health literacy			Healthcare			Disease prevention			Health promotion		
	M (SD)	F/t	*p*	M (SD)	F/t	*p*	M (SD)	F/t	*p*	M (SD)	F/t	*p*
Sex												
Male	3.58 (0.70)	−0.41	0.68	3.55 (0.71)	−0.22	0.83	3.56 (0.82)	−0.66	0.51	3.67 (0.75)	−0.30	0.77
Female	3.61 (0.76)			3.56 (0.78)			3.61 (0.84)			3.69 (0.82)		
Region												
Highlands	3.51 (0.73)	7.33	<0.01*	3.47 (0.74)	6.57	<0.01*	3.50 (0.84)	6.14	<0.01*	3.60 (0.81)	6.26	<0.01*
Coast	3.79 (0.68)			3.74 (0.70)			3.79 (0.80)			3.88 (0.70)		
Jungle	3.47 (0.72)			3.47 (0.74)			3.44 (0.78)			3.50 (0.75)		
Department												
Junín	3.50 (0.71)	1.75	0.14	3.47 (0.72)	1.54	0.19	3.49 (0.83)	1.58	0.18	3.57 (0.78)	2.03	0.09
La Libertad	3.73 (0.56)			3.62 (0.59)			3.78 (0.70)			3.87 (0.63)		
Arequipa	3.61 (0.69)			3.55 (0.68)			3.62 (0.75)			3.71 (0.78)		
Lima	3.75 (0.67)			3.77 (0.66)			3.69 (0.78)			3.78 (0.72)		
Other	3.62 (0.83)			3.59 (0.86)			3.59 (0.93)			3.72 (0.85)		
Ethnicity												
Mestizo	3.62 (0.77)	0.99	0.39	3.57 (0.78)	0.70	0.55	3.62 (0.88)	1.28	0.28	3.70 (0.81)	0.73	0.53
Quechua	3.50 (0.63)			3.46 (0.66)			3.46 (0.71)			3.60 (0.71)		
Caucasian	3.73 (0.59)			3.68 (0.54)			3.72 (0.74)			3.83 (0.70)		
Other	3.48 (0.46)			3.50 (0.49)			3.39 (0.54)			3.57 (0.64)		
Educational level												
Superior university	3.69 (0.67)	6.75	<0.001*	3.64 (0.67)	6.88	<0.01*	3.68 (0.77)	5.06	<0.01*	3.77 (0.73)	5.31	<0.001*
Technical superior	3.60 (0.68)			3.59 (0.72)			3.56 (0.80)			3.68 (0.75)		
High School	3.52 (0.81)			3.48 (0.82)			3.52 (0.91)			3.60 (0.84)		
Incomplete Elementary School	2.98 (0.78)			2.91 (0.77)			2.98 (0.89)			3.11 (0.93)		
Housing												
Own	3.61 (0.69)	0.81	0.42	3.57 (0.70)	0.58	0.56	3.60 (0.80)	0.75	0.45	3.71 (0.75)	1.04	0.30
Rented	3.55 (0.79)			3.52 (0.81)			3.54 (0.89)			3.62 (0.86)		
Government social support												
No	3.59 (0.72)	−0.17	0.86	3.55 (0.74)	−0.58	0.57	3.58 (0.82)	0.02	0.98	3.68 (0.78)	0.22	0.83
Yes	3.62 (0.79)			3.63 (0.75)			3.58 (0.96)			3.65 (0.85)		
Employment status												
Dependent worker	3.54 (0.66)	1.08	0.34	3.55 (0.66)	0.04	0.97	3.49 (0.80)	2.59	0.08	3.60 (0.73)	2.02	0.13
Self-employed	3.60 (0.74)			3.55 (0.77)			3.59 (0.84)			3.70 (0.79)		
Unemployed	3.67 (0.80)			3.57 (0.82)			3.72 (0.85)			3.79 (0.84)		
Time to get to work												
Less than 15 min	3.72 (0.66)	3.50	0.02	3.70 (0.67)	2.88	0.04	3.69 (0.79)	3.48	0.02	3.80 (0.72)	3.66	0.01
15 to 30 min	3.46 (0.76)			3.42 (0.78)			3.45 (0.86)			3.53 (0.82)		
More than 30 min	3.50 (0.66)			3.49 (0.68)			3.45 (0.79)			3.57 (0.73)		
Unemployed	3.67 (0.80)			3.57 (0.84)			3.72 (0.85)			3.79 (0.84)		
Working hours per week												
Less than 24 h	3.64 (0.75)	2.47	0.06	3.58 (0.76)	1.27	0.29	3.65 (0.86)	3.49	0.02	3.73 (0.84)	2.84	0.04
24 to 48 h	3.65 (0.69)			3.62 (0.72)			3.62 (0.80)			3.73 (0.84)		
More than 48 h	3.46 (0.68)			3.45 (0.68)			3.40 (0.80)			3.52 (0.73)		
Unemployed	3.67 (0.80)			3.57 (0.82)			3.72 (0.85)			3.79 (0.84)		
Financial income												
Do not meet their needs	3.46 (0.81)	15.05	<0.001*	3.39 (0.83)	15.42	<0.001*	3.45 (0.92)	10.04	<0.001*	3.59 (0.87)	14.22	<0.001*
They cover your needs, but you cannot save	3.52 (0.67)			3.50 (0.69)			3.51 (0.77)			3.58 (0.74)		
They meet your needs and can also save you money	3.93 (0.65)			3.89 (0.65)			3.91 (0.79)			4.03 (0.69)		
Health insurance												
SIS	3.51 (0.76)	4.87	<0.01	3.49 (0.75)	4.34	<0.01	3.50 (0.86)	4.03	<0.01	3.56 (0.84)	5.23	<0.01
EsSalud	3.61 (0.65)			3.58 (0.67)			3.57 (0.79)			3.70 (0.70)		
None	3.60 (0.80)			3.48 (0.88)			3.63 (0.89)			3.77 (0.78)		
Private	4.00 (0.51)			3.94 (0.54)			4.01 (0.55)			4.08 (0.63)		
Time to go to a health care facility												
Less than 15 min	3.75 (0.72)	4.99	<0.01	3.72 (0.75)	5.71	<0.01	3.74 (0.85)	3.95	<0.01	3.82 (0.75)	3.25	0.02
15 to 30 min	3.64 (0.70)			3.62 (0.69)			3.62 (0.80)			3.72 (0.78)		
30 to 45 min	3.48 (0.59)			3.39 (0.61)			3.53 (0.71)			3.57 (0.69)		
More than 45 min	3.39 (0.85)			3.36 (0.88)			3.33 (0.94)			3.51 (0.90)		
Chronic disease												
No	3.60 (0.73)	0.24	0.79	3.56 (0.74)	0.22	0.80	3.59 (0.84)	0.34	0.71	3.69 (0.80)	0.33	0.72
Yes, one	3.55 (0.62)			3.55 (0.71)			3.49 (0.76)			3.64 (0.65)		
Yes, more than one	3.47 (0.80)			3.40 (0.93)			3.54 (0.79)			3.50 (0.76)		
Caregiver of chronically ill family member												
No	3.60 (0.73)	0.02	0.99	3.57 (0.74)	0.01	1.00	3.59 (0.82)	0.19	0.83	3.68 (0.79)	0.08	0.92
Yes, one	3.57 (0.78)			3.55 (0.84)			3.50 (1.01)			3.71 (0.68)		
Yes, more than one	3.59 (0.42)			3.57 (0.31)			3.62 (0.49)			3.60 (0.79)		

*ANOVA.

Significant differences in HL levels were observed based on educational attainment (*F* = 6.75; *p* < 0.001) (Table 4). Post-hoc analysis indicated that participants with primary or incomplete education had significantly lower HL levels (mean = 2.98) compared to those with secondary education (mean = 3.53; *p* = 0.01), higher technical education (mean = 3.61; *p* < 0.01), and university education (mean = 3.70; *p* < 0.001). This difference was especially pronounced in the “healthcare” domain (*F* = 6.88; *p* < 0.001).

The time spent commuting to work also showed statistically significant differences across groups (*F* = 3.50; *p* = 0.02). Participants with a commute of less than 15 min had higher health literacy scores (mean = 3.72) compared to those with longer commute times (Table 4).

Workload was moderately associated with health literacy in the domains of “disease prevention” (*F* = 3.49; *p* = 0.02) and “health promotion” (*F* = 2.84; *p* = 0.04) (Table 4). Post-hoc analysis revealed that participants working more than 48 h per week had lower scores in both domains (mean = 3.40 for “disease prevention”; mean = 3.52 for “health promotion”) compared to unemployed participants (mean = 3.72 for “disease prevention”; mean = 3.79 for “health promotion”), suggesting that the unemployed group had higher levels of HL in both domains.

Economic income also showed significant differences in HL levels (*F* = 14.04; *p* < 0.001) across all three domains, with the most notable difference found in the “healthcare” domain (*F* = 13.96; *p* < 0.001). Participants whose income was sufficient to meet their needs and save had significantly higher HL scores (mean = 3.93). Scores were lower among those whose income covered only basic needs but did not allow saving (mean = 3.52; *p* < 0.001) and those whose income did not cover their needs (mean = 3.46; *p* < 0.001) (Table 4).

Likewise, significant differences were observed based on health insurance type (*F* = 4.87; *p* < 0.01). Participants with private insurance had significantly higher levels of HL (mean = 4.00) compared to those with SIS (mean = 3.51; *p* < 0.01), EsSalud (mean = 3.61; *p* = 0.02) or no insurance (mean = 3.60; *p* = 0.04).

Proximity to health institutions showed a significant impact on participants’ HL levels (*F* = 4.99; *p* < 0.01) (Table 4). *Post hoc* analyses revealed that participants who took less than 15 min to reach a healthcare facility had significantly higher HL levels (mean = 3.75) than to those who took 30–45 min (mean = 3.48; *p* = 0.04) or more than 45 min (mean = 3.39; *p* < 0.01). The differences were statistically significant across all three domains, being most pronounced in the “healthcare” domain (*F* = 5.71; *p* < 0.01).

Age was negatively correlated with HL, with a small but statistically significant relationship (*r* = −0.13; *p* < 0.001), suggesting that higher age is associated with lower HL. This negative association was significant across all three domains and slightly more pronounced in the “disease prevention” domain (*r* = −0.14; *p* < 0.01) (Table 5).

**Table 5:** Numerical characteristics of the population by HL and its dimensions.

Characteristic	Health Literacy		Healthcare		Disease prevention		Health promotion	
	*r*	p*	*r*	p*	*r*	p*	*r*	p*
Age	−0.13	0.01	−0.11	0.02	−0.14	0.00	−0.11	0.03
Time chronic disease	−0.15	0.03	−0.02	0.88	−0.25	0.08	−0.15	0.29
Time as caregiver	−0.12	0.45	−0.01	0.95	−0.12	0.43	−0.22	0.15

^*^Pearson’s correlation.

A supplementary analysis using the original dichotomous scoring method of the HLS-EU-Q16 was conducted (Table 6). Chi-square tests (or Fisher’s exact test when expected cell frequencies were <5) examining associations between sociodemographic variables and categorical HL levels (Inadequate, Problematic, Sufficient) showed partial convergence with ANOVA results. Five variables were significant in both approaches: region (χ^2^ = 11.938, *p* = 0.018), financial income (χ^2^ = 25.071, *p* < 0.001), health insurance (χ^2^ = 18.866, *p* = 0.004), and time to healthcare facility (χ^2^ = 15.181, *p* = 0.019), demonstrating robust associations regardless of analytical method. However, discordant patterns emerged for several variables. Region of residence showed significant categorical associations (χ^2^ = 19.086, *p* = 0.014) despite non-significant ANOVA results, with “Other” regions exhibiting notably higher Sufficient HL proportions (40.9%). Conversely, educational level (Fisher’s exact test, *p* = 0.251), time to work, and working hours per week showed significant ANOVA effects but non-significant categorical tests, suggesting these variables produce subtle continuous differences insufficient to generate distinct categorical shifts. Variables including sex (χ^2^ = 1.333, *p* = 0.513), ethnicity (Fisher’s exact test, *p* = 0.31), housing (χ^2^ = 1.338, *p* = 0.512), employment status (χ^2^ = 2.566, *p* = 0.633), and health conditions (Fisher’s exact test for chronic disease, *p* = 0.40; caregiver status, *p* = 0.725) remained non-significant across both approaches.

**Table 6:** Supplementary analysis.

Characteristics	Inadequate M (SD)	Problematic M (SD)	Sufficient M (SD)	Chi-square	*p*-value
Sex					
Male	108 (44.8)	58 (24.1)	75 (31.1)	1.33	0.51
Female	87 (44.2)	40 (20.3)	70 (35.5)		
Region					
Highlands	138 (49.5)	57 (20.4)	84 (30.1)	11.94	0.02
Coast	42 (32.1)	36 (27.5)	53 (40.5)		
Jungle	15 (53.6)	5 (17.9)	8 (28.6)		
Department					
Junin	94 (51.4)	40 (21.9)	49 (26.8)	19.09	0.01
La Libertad	18 (34.0)	19 (35.8)	16 (30.2)		
Arequipa	22 (43.1)	9 (17.6)	20 (39.2)		
Lima	11 (30.6)	12 (33.3)	13 (36.1)		
Other	50 (43.5)	18 (15.7)	47 (40.9)		
Ethnicity					
Mestizo	136 (42.6)	69 (21.6)	114 (35.7)	–	0.31
Quechua	40 (51.9)	18 (23.4)	19 (24.7)		
Caucasian	6 (31.6)	6 (31.6)	7 (36.8)		
Other	13 (56.5)	5 (21.7)	5 (21.7)		
Educational level					
Incomplete Elementary School	14 (66.7)	4 (19.0)	3 (14.3)	–	0.25
High School	45 (44.6)	27 (26.7)	29 (28.7)		
Technical superior	47 (46.1)	22 (21.6)	33 (32.4)		
Superior university	89 (41.6)	45 (21.0)	80 (37.4)		
Housing					
Own	131 (44.4)	62 (21.0)	102 (34.6)	1.34	0.51
Rented	64 (44.8)	36 (25.2)	43 (30.1)		
Government social support					
No	182 (44.5)	93 (22.7)	134 (32.8)	0.59	0.75
Yes	13 (44.8)	5 (17.2)	11 (37.9)		
Employment status					
Dependent worker	84 (48.6)	34 (19.7)	55 (31.8)	2.57	0.63
Self-employed	68 (42.5)	40 (25.0)	52 (32.5)		
Unemployed	43 (41.0)	24 (22.9)	38 (36.2)		
Working hours per week					
Less than 24 h	28 (44.4)	8 (12.7)	27 (42.9)	7.97	0.24
24 to 48 h	61 (43.3)	34 (24.1)	46 (32.6)		
More than 48 h	63 (48.8)	32 (24.8)	34 (26.4)		
Unemployed	43 (41.0)	24 (22.9)	38 (36.2)		
Time to get to work					
< 15 min	47 (38.2)	28 (22.8)	48 (39.0)	9.93	0.13
15–30 min	52 (54.7)	15 (15.8)	28 (29.5)		
> 30 min	53 (46.1)	31 (27.0)	31 (27.0)		
Unemployed	43 (41.0)	24 (22.9)	38 (36.2)		
Financial income					
Do not meet their needs	58 (54.2)	18 (16.8)	31 (29.0)	25.07	<0.001
They cover your needs, but you cannot save	114 (48.1)	57 (24.1)	66 (27.8)		
They meet your needs and can also save you money	23 (24.5)	23 (24.5)	48 (51.1)		
Health insurance					
EsSalud	63 (44.4)	29 (20.4)	50 (35.2)	18.87	0.004
None	33 (52.4)	8 (12.7)	22 (34.9)		
Private	6 (16.2)	12 (32.4)	19 (51.4)		
SIS	93 (47.4)	49 (25.0)	54 (27.6)		
Time to go to a health care facility					
< 15 min	41 (34.7)	30 (25.4)	47 (39.8)	15.18	0.019
15–30 min	69 (45.4)	27 (17.8)	56 (36.8)		
30–45 min	46 (50.0)	27 (29.3)	19 (20.7)		
> 45 min	39 (51.3)	14 (18.4)	23 (30.3)		
Chronic disease					
No	172 (44.6)	86 (22.3)	128 (33.2)	–	0.40
Yes, one	17 (40.5)	12 (28.6)	13 (31.0)		
Yes, more than one	6 (60.0)	0 (0.0)	4 (40.0)		
Caregiver of chronically ill family member					
No	176 (44.7)	86 (21.8)	132 (33.5)	–	0.73
Yes, one	15 (44.1)	8 (23.5)	11 (32.4)		
Yes, more than one	4 (40.0)	4 (40.0)	2 (20.0)		

These findings suggest that while most associations are consistent across analytical methods, categorization may enhance detection of non-linear patterns (e.g., region) while reducing sensitivity to modest linear effects (e.g., education, work-related variables).

## 4 Discussion

The study assessed the psychometric properties of the short-form HLS-EU-Q16 questionnaire, administered in Spanish, among Peruvian adults. The assessment comprised adapting the instrument to the Peruvian context and measuring its construct validity, reliability, and sensitivity.

### 4.1 Cultural adaptation

The cultural adaptation process helped to identify regional language variations. Specifically, the words “pharmacist” and “Pilates” were found to be less commonly used in Peru, which could lead to confusion. Adding “health professionals” broadened the scope of responses. These adjustments ensured that the questionnaire was syntactically and grammatically appropriate for the target population. A thorough, context-specific review is essential when adapting instruments originally developed in other languages, as it facilitates the proper framing of questions for the target population and ensures acceptance (9). The adaptation also demonstrated high internal consistency, consistent with findings from other adaptability studies (9, 24).

### 4.2 Validity and reliability

Construct validity was evaluated through factor analysis of a three-dimensional model: healthcare, disease prevention, and health promotion (12). The analysis demonstrated a coherent structure, with items logically grouped within each domain, adequately reflecting the previously defined theoretical concepts. The fit of the model to the data indicates that it adequately represents the measured reality, reinforcing its construct validity. The analysis confirmed that the correlations observed between items were not random but aligned with key aspects of the construct being studied (34). Furthermore, the difference between the three domains suggests that the questionnaire can capture important nuances in assessing variables related to healthcare, disease prevention, and health promotion. This multidimensional structure enables a more comprehensive interpretation of the results.

Our decision to use continuous scoring rather than the original dichotomous method was guided by established psychometric principles. Polytomous response formats allow greater variability and precision in measurement compared to dichotomous formats and are more amenable to various statistical analyses (35). Dichotomizing continuous variables reduces statistical power by an amount equivalent to discarding one-third of the data and may obscure non-linear relationships (36). Recent international validations of the HLS-EU-Q16 have increasingly adopted continuous scoring for psychometric analyses (24), while recognizing that dichotomous scoring remains valuable for clinical screening applications where categorical risk stratification is needed.

The reliability of the questionnaire was assessed using Cronbach’s alpha and the omega coefficient. Both measures indicated excellent internal consistency, suggesting that the culturally adapted instrument reliably measures the construct. These results indicate a high level of homogeneity and reliability across the overall scale and each domain.

Overall, these results confirm that the scores are stable and reproducible (25, 34), reinforcing the instrument’s utility. Moreover, it accurately measures the intended concepts and provides reliable results across all its domains.

### 4.3 Socio-demographic characteristics and health literacy levels of the Peruvian population

The study found statistically significant differences in health literacy (HL) levels associated with key sociodemographic factors such as geographic region. Participants from the coast exhibited significantly higher HL levels than those from the highlands. Educational attainment also played a significant role; participants with lower educational attainment—such as those who completed only primary school or had incomplete secondary education—demonstrated significantly lower HL levels, particularly in the healthcare domain. Commute time was another influential factor: participants whose commute lasted less than 15 min had higher HL levels than those with longer commute times. Hours worked per week also significantly impacted HL levels, especially in the disease-prevention and health-promotion domains; unemployed participants exhibited higher levels in these domains than those who worked more than 48 h per week. Regarding income, participants who had sufficient income to meet their needs and were able to save reported significantly higher HL levels compared with those whose income only covered basic necessities or was insufficient; this difference was most pronounced in the healthcare domain.

In summary, differences in HL are influenced by region, educational attainment, commute time, hours worked, and income. These findings demonstrate how socioeconomic and labour conditions influence individuals’ ability to process and apply health-related information. The results are consistent with prior studies demonstrating that the HLS-EU-Q16 distinguishes between HL levels according to sociodemographic factors and social inequalities (7).

The differences in HL between the coastal and highland regions can be partly attributed to social, economic, and educational factors that vary between geographic areas. Previous studies, such as those by Sørensen et al. (20), have indicated that disparities in HL are related to financial deprivation and unequal access to resources, suggesting a social gradient in health. This reinforces the importance of adapting measurement tools to specific contexts so that these differences can be captured accurately, as highlighted by Gustafsdottir et al. (9). In Chile, Figueroa et al. (37) found that lower educational levels were associated with poorer HL, a finding consistent with ours and underscoring the crucial role of education in understanding and applying health information. These findings support the need for health policies that consider regional differences, promoting strategies that reduce inequities and improve HL equitably.

Additionally, several studies have demonstrated that educational level influences health literacy (7, 11, 34, 38–41), suggesting that higher educational attainment is associated with a greater capacity to access, understand, interpret, evaluate health information, make informed health decisions, and follow medical advice.

Health literacy is also influenced by income. Participants with sufficient financial resources to cover their needs experience less financial stress, leading to lower levels of anxiety (25, 40, 41). Having health insurance is also associated with higher HL levels (7). However, it is important to note that in Peru, both the SIS and EsSalud systems face challenges –such as management issues, structural problems, medication shortages, and corruption (42–44)– and the complexity of the health-care system (45), may influence HL levels. This study did not measure these factors and further research is needed.

The study also found that participants with easier access to health services and shorter commute times had higher HL levels, which is consistent with previous research by Mialhe et al. (25). Their findings suggest that easier access to services promotes a better understanding and use of health information. Furthermore, the relationship between shorter work hours—or unemployment status—and higher HL levels may be attributed to the increased time available for accessing health resources and participating in educational activities. Oliva Ramírez (46) emphasizes that health empowerment is conditioned by time and available resources, reinforcing the idea that work conditions and healthcare accessibility are key determinants of health literacy. These findings underscore the importance of considering living and working conditions when designing strategies to improve health literacy.

Finally, the study found that older the participants tended to have lower HL levels, as in other studies (34, 38–41).

### 4.4 Psychometric properties of the HLS-EU-Q16 questionnaire

The confirmatory factor analysis conducted in Peru validated the three-dimensional model of the HLS-EU-Q16. The fit indicators showed optimal values, and all factor loadings were positive and above the threshold, supporting the instrument’s theoretical structure in this population. In addition, the high and positive correlations between domains suggest a strong interrelationship between them. These findings confirm the validity of the model in the Peruvian context and are consistent with studies conducted in Portugal (23) and Venezuela (31). However, they differ from results found in Mexico (22), Iceland (9), and Spain (21), where different factor structures were reported. These variations reflect how HL domains may vary across cultures and contexts, highlighting the need to adapt and validate instruments in each specific population.

Internal consistency was high across all dimensions, as indicated by Cronbach’s alpha and the omega coefficient. These results are comparable to those obtained in Portugal. (23), and Mexico (22) and slightly higher than those reported in France and Romania (20). These values support the reliability of the adapted instrument for measuring health literacy in the Peruvian context.

### 4.5 Public health implications

The validated HLS-EU-Q16 offers multiple practical applications for Peru’s health system. First, it serves as a surveillance tool for monitoring HL levels across diverse populations and geographic regions, enabling the identification of vulnerable groups that require targeted support. The instrument’s brief format (16 items) makes it feasible for large-scale population surveys and routine assessment in primary health-care settings.

Second, the HLS-EU-Q16 can inform health policy planning and resource allocation. Our findings reveal significant disparities in health literacy based on region (coast versus highlands), educational level, income, and health-care access. These results can guide policymakers in direct resources toward highland and rural regions, populations with lower educational attainment, and communities with limited health-care access.

Third, the instrument enables design and evaluation of targeted interventions. Health-care facilities can use the HLS-EU-Q16 to screen patients, identify those with low HL in specific domains (healthcare, disease prevention, health promotion), and provide tailored health-education materials and communication strategies. The three-dimensional structure allows practitioners to determine whether individuals struggle more with navigating health-care systems, preventing disease, or promoting health; this facilitates domain-specific interventions.

Finally, the instrument can support health-equity initiatives by providing objective data on HL inequalities. Regular monitoring with the HLS-EU-Q16 can track progress in reducing HL gaps and evaluate the effectiveness of national health-literacy improvement programs.

### 4.6 Limitations

While the results are robust, several important limitations should be considered when interpreting our findings.

First, sampling limitations: This study employed non-probability convenience sampling, which limits the representativeness and generalizability of findings. The recruitment strategy and online survey format over-represent more educated, urban, and digitally connected participants with better access to technology and internet connectivity. Rural populations, individuals with lower educational levels, and those with limited digital literacy may be underrepresented. Future research should employ probability-based sampling methods (e.g., stratified random sampling) to ensure representative coverage of Peru’s diverse population, including hard-to-reach rural and Indigenous communities.

Second, measurement approach: Our deviation from the original dichotomous scoring method in favor of continuous scoring—while justified by psychometric principles and supported by supplementary analysis (Table 6)—may limit direct comparability with studies using the standard HLS-EU categorization. Additionally, the use of self-administered online questionnaires may introduce response biases such as social desirability and acquiescence bias, potentially affecting response accuracy.

Third, analytical limitations: Our analysis relied primarily on univariate statistical methods (ANOVA, t-tests, correlations) to examine sociodemographic differences. While these methods appropriately address our study objectives, they do not account for potential confounding effects or identify independent predictors of HL. Future research should employ multivariable regression modeling to simultaneously control multiple sociodemographic factors and determine which variables exert independent effects on HL. Additionally, effect sizes and confidence intervals should be reported to enhance interpretability beyond statistical significance.

Fourth, factorial invariance: The study did not assess measurement invariance across different geographic regions, ethnic groups, or other sociodemographic subgroups. Without establishing invariance, we cannot definitively conclude that the instrument functions equivalently across Peru’s diverse populations. Future research should conduct multi-group confirmatory factor analysis to test configural, metric, and scalar invariance across key subgroups, ensuring fair comparisons.

Fifth, cross-sectional design: The cross-sectional nature of this study precludes causal inferences about the relationships between sociodemographic factors and health literacy. Longitudinal studies are needed to examine how health literacy changes over time, identify causal pathways, and evaluate the impact of interventions on health literacy improvement.

Despite these limitations, this study provides the first validated assessment of the HLS-EU-Q16 in Peru with a large, geographically diverse sample. The findings offer valuable insights into HL patterns and establish a foundation for future research addressing the methodological limitations identified above.

### 4.7 Conclusion

This study provides the first validated Spanish version of the HLS-EU-Q16 for Peru, demonstrating excellent psychometric properties in a diverse sample of Peruvian adults. The three-dimensional structure (health care, disease prevention, and health promotion) was confirmed with strong internal consistency and discriminant validity across sociodemographic groups. Significant health literacy disparities were identified based on geographic region, educational level, income, and access to health services, with coastal populations, individuals with higher education, and those with greater economic stability showing higher health literacy levels. These findings highlight substantial inequalities in the ability to access, understand, and use health information across Peru. The validated HLS-EU-Q16 provides researchers and public health practitioners with a reliable, culturally appropriate tool to assess health literacy and design targeted interventions to reduce health inequities in the Peruvian population. Future research should focus on developing evidence-based strategies to improve health literacy, particularly among vulnerable populations in highland and rural regions, those with lower educational attainment, and individuals facing economic constraints or limited access to health services.

### 4.8 Contribution to the field

Many Peruvians struggle to find, understand, and use health information, but Peru lacks a validated tool to measure this problem. This study provides the first reliable questionnaire to assess health literacy across Peru’s diverse population. We found significant gaps: people in coastal regions with higher levels of education, higher incomes, and easier access to health services understood health information better than those in highlands who have less education, lower incomes, or lived far from health facilities. These gaps help explain health inequalities across Peru. This tool enables public health officials to identify communities that need support, measure program effectiveness, and design targeted interventions to reduce health inequities. By revealing where health literacy problems exist, this research provides a foundation for fairer health programs that reach all Peruvians, especially the most vulnerable populations.

## Data Availability

The datasets presented in this study can be found in online repositories. The names of the repository/repositories and accession number(s) can be found at: https://doi.org/10.6084/m9.figshare.28544561.
