# Correction: Health literacy: psychometric properties of the HLS-EU-Q16 questionnaire in a Peruvian adult population

**DOI:** 10.3389/fpubh.2026.1830301

**Published:** 2026-04-02

**Authors:** Daniel F. Condor-Camara, Judith Francisco-Pérez, Gabriel Ortiz-Francisco, Zoila Esperanza Leiton-Espinoza, Johnny R. Jurado Boza, Nancy Ana Coaquira Machaca, Katherine L. Sánchez-Álvarez, Yuly S. Hilario-Pizarro, Jacqueline Begazo Corahua, Nataly Ventura-Román

**Affiliations:** 1 Faculty of Nursing, Universidad Peruana Cayetano Heredia, Lima, Peru; 2 Faculty of Health and Wellbeing, Pontificia Universidad Católica del Ecuador, Quito, Ecuador; 3 Faculty of Psychology, Universidad Técnica Particular de Loja, Loja, Ecuador; 4 Faculty of Nursing, Universidad Nacional de Trujillo, Trujillo, Peru; 5 Faculty of Nursing, Universidad Peruana Los Andes, Huancayo, Peru; 6 Faculty of Nursing, Universidad Nacional de San Agustin de Arequipa, Arequipa, Peru; 7 Faculty of Medicine, Postgraduate School, Universidad Nacional Mayor de San Marcos, Lima, Peru; 8 Faculty of Health Sciences, Universidad Nacional Daniel Alcides Carrion, Tarma, Peru; 9 Challhuani Health Center, Regional Health Directorate of Apurimac, Challhuani, Apurimac, Peru

**Keywords:** global health, health education, health literacy, primary health care, public health

An incorrect **Funding** statement was provided. It originally stated: “The author(s) declared that financial support was not received for this work and/or its publication.”

The correct **funding** statement reads:

“The author(s) declared that financial support was received for this work and/or its publication. This work was funded by the Universidad Peruana Cayetano Heredia, ID 212114.”

The original version of this article has been updated.

